# Dietary Effects of Probiotic Bacteria, *Bacillus amyloliquefaciens* AV5 on Growth, Serum and Mucus Immune Response, Metabolomics, and Lipid Metabolism in Nile Tilapia (*Oreochromis niloticus*)

**DOI:** 10.1155/2024/4253969

**Published:** 2024-08-12

**Authors:** Vicent Michael Shija, Glory Emanuel Zakaria, Kwaku Amoah, Li Yi, Junwei Huang, Fortunatus Masanja, Zhong Yong, Jia Cai

**Affiliations:** ^1^ College of Fishery Guangdong Ocean University, Zhanjiang 524088, China; ^2^ Guangdong Provincial Key Laboratory of Aquatic Animal Disease Control and Healthy Culture, Zhanjiang 524088, China; ^3^ Guangdong Key Laboratory of Control for Diseases of Aquatic Economic Animals, Zhanjiang 524088, China; ^4^ Southern Marine Science and Engineering Guangdong Laboratory, Zhanjiang 524002, China; ^5^ Guangxi Key Laboratory for Marine Natural Products and Combinational Biosynthesis Chemistry, Nanning 530200, China; ^6^ Guangxi Beibu Gulf Marine Research Centre Guangxi Academy of Sciences, Nanning 530007, China

## Abstract

In the present study, we investigated the effect of dietary supplementation with the probiotic *Bacillus amyloliquefaciens* AV5 (OR647358) on the growth, serum and mucus immune responses, metabolomics, and lipid metabolism of *Oreochromis niloticus*. Fishes (27.2 ± 1.7 g and 9.0 ± 1.2 cm) were fed three distinct meals: a commercial diet (control-GC) and two treatment diets supplemented with probiotics at 10^6^ (G1) and 10^8^ cfu/g (G2), respectively, for 30 days. In the G2 group, the final weight, specific growth rate, weight gain rate, survival rate, and feed conversion ratio of the fish were significantly improved (*p* < 0.05). Lysozyme, myeloperoxidase, and alkaline phosphatase activities in the mucus of fish were significantly higher (*p* < 0.05) in the G1 and G2 groups. The serum total protein, superoxide dismutase, glutathione peroxidase, reactive oxygen species, and reactive nitrogen species levels were noticeably higher (*p* < 0.05) in fish fed G1 and G2. In addition, in the G1 and G2 groups, higher levels of enzymes involved in lipid metabolism, such as pyruvate kinase, 2-hydroxyethyl-ThPP, and dihydrolipoamide dehydrogenase, were increased. Distal gastrointestinal metabolites, such as glycerophospholipids and histidine, were observed. These findings strongly indicate that incorporating *B. amyloliquefaciens* AV5 at 10^8^ cfu/g into commercial feeds positively influences fish growth, immunity, and lipid metabolism.

## 1. Introduction

In recent years, the increased production of Nile tilapia (*Oreochromis niloticus*) has been accompanied by an increase in infections and disease outbreaks, causing significant economic losses in the aquaculture industry [1]. Probiotics are a promising remedy for aquaculture, offering many advantages, such as enhancing fish growth, preserving the environment, and bolstering disease resilience, compared with alternative preventive approaches [2, 3]. Potential probiotic strains exhibit diverse characteristics that enable them to endure challenging conditions in the gastrointestinal tract. These traits include acid and bile salt tolerance, resilience against gastric juices, production of extracellular enzymes and antimicrobial substances capable of eliminating or inhibiting pathogen growth, adherence to intestinal mucus, and the capacity to reside in the gut [4]. Moreover, probiotic strains must fulfill biosafety criteria including nonhemolytic activity and antibiotic susceptibility [4]. Probiotic *Bacillus* species exhibit a remarkable array of mechanisms to inhibit fish pathogens, ranging from direct antibacterial actions such as bacteriocin production and lytic enzyme activity to indirect strategies such as immunostimulation and competitive exclusion [5]. *Bacillus* spp. are unique probiotics because they can produce spores. These spores produce nonpathogenic and nontoxic compounds, improve water quality, and make bacteria resistant to unfavorable conditions [4]. These characteristics distinguish them from other probiotics and provide advantages such as heat tolerance and extended shelf life [6]. Many studies have shown that certain types of *Bacillus* species are useful in aquaculture, particularly in Nile tilapia farming [7, 8, 9]. Catfish fed *Bacillus amyloliquefaciens* can boost their immune system [10]. *Bacillus amyloliquefaciens* YL-10 has the potential to be employed as a probiotic bacterium to prevent vibriosis infections in *Haliotis discus hannai* [11]. In a study by Liu et al. [12], adding *B. subtilis* E20 to the diet improved immune cells during phagocytosis and made *Oplegnathus fasciatus* less likely to cause infections. Metabolomics is a scientific discipline that helps uncover changes in metabolism by analyzing small molecules in biofluids, tissues, and organs, which play crucial roles in all biological processes [13, 14]. Nevertheless, the optimal levels at which *B. amyloliquefaciens* should be added to the diet of freshwater Nile tilapia to enhance immunological responses, metabolite levels, and lipid metabolism remain unclear [15, 16, 17]. This study investigated the impact of feed with varying levels of *B. amyloliquefaciens* AV5 on the growth, immune response, metabolomics, and lipid metabolism of Nile tilapia (*O. niloticus*).

## 2. Materials and Methods

### 2.1. Animal Ethics

The experiments were done according to the guidelines for the care and use of laboratory animals in China. The research received approval from the Ethics Committee for Animal Experiments at Guangdong Ocean University.

### 2.2. Experimental Fish and Rearing Condition

The healthy Nile tilapia fingerlings, *O. niloticus* (weight, 23.0 ± 1.5 g and length, 8.0 ± 1.2 cm), were sourced from Langye fish farm in Gaozhou, Guangdong province, China. These fingerlings exhibited no signs of bleeding, lethargy, ascites, or detachment of scales. They were transported and placed in plastic tanks at the Key Laboratory of Aquatic Animal Disease Control and Healthy Culture at Guangdong Ocean University. The fishes were given a 14-day period to acclimate before the commencement of the experimental study. The fishes were given commercial feed (Guangdong Yuehai Feed Group, Zhanjiang, China) twice daily, equivalent to 3% of their body weight. The fish's weight was measured weekly to evaluate growth and implement any required adjustments to the daily feeding ratio. Excrement and unconsumed food were periodically removed via siphoning. To prevent stress, the researchers consistently delivered aeration throughout the acclimation phase. Throughout this time frame, their health conditions were observed and assessed according to the study performed by Gobi et al. [18]. The water quality metrics were maintained at their basic levels throughout the probation and experimental trial periods, following the procedure undertaken by de Verdal et al. [19], with slight adjustments. Briefly, the conditions were as follows: temperature, 28.0 ± 1.5°C; dissolved oxygen, 6.8 ± 0.9 mg/L; pH level, 6.9 ± 1.2; and photoperiod, 13 hr of light followed by 11 hr of dark.

### 2.3. Strain Conditions

The strain *B. amyloliquefaciens* AV5 (OR647358) was obtained from the intestines of Nile tilapia in their natural habitat, and its molecular identity was determined using 16S rDNA gene sequencing (NCBI GenBank accession number OR647358). Previous studies have suggested that *B. amyloliquefaciens* has probiotic properties [20, 21]. The probiotic *B. amyloliquefaciens* AV5 used in this work was obtained from our laboratory's stock culture collection. It was preserved at −80°C in a 40% v/v solution of sterile glycerol in Luria–Bertani (LB) broth. To perform regular experiments, researchers obtained a preserved strain culture from the stocks kept at −80°C. This culture was then spread on nutrient agar and incubated overnight at 37°C in the presence of oxygen. We used a shaker incubator to cultivate a single isolated colony in 100 mL LB broth for 24 hr at 37°C. The cell density was determined by measuring the optical density at 600 nm (OD600) and then comparing it to the number of colony-forming units (cfu) obtained by serial dilution and spread plating on nutritional agar. The bacterial biomass was collected and centrifuged at 8,000 *g* for 20 min at 4°C. The liquid portion (supernatant) was extracted, and the bacteria pellets were diluted again in PBS at 10^6^ and 10^8^ cfu/mL.

### 2.4. Diets Preparation

Following the nutritional requirements of Nile tilapia, a basal diet from Guangdong Yuehai Feed Group (Zhanjiang, China) was used in the experiment.  Table 1 displays the main ingredients and approximate composition of the basal diet according to the manufacturer's information. In the treatment trial, two different concentrations (10^6^ and10^8^ cfu/g) of probiotic *B. amyloliquefaciens* AV5 were added to the basal diet. The concentrations were chosen according to the studies conducted by Selim and Reda [22] and Thy et al. [23] with slight modifications. Briefly, probiotic suspensions were progressively sprayed (80 mL probiotic suspension/kg feed) on the commercial feed (Guangdong Yuehai Feed Group, Zhanjiang, China) before being blended gently using a mixer. The diets were dehydrated at 38°C in a drying cabinet and kept at 4°C until they were used again. The weekly assessment of the survivability of bacteria added to the diet was conducted using the technique developed by Abarike et al. [24], with minor adjustments. In a nutshell, 1.0 g of feed was thoroughly mixed with 9.0 mL of sterile saline solution (PBS), and further solutions were made in a series of dilutions up to 10^5^. One hundred microliters of each was evenly inoculated into nutrient agar dishes in triplicate, incubated at 33°C for 24 hr, and the colonies were then determined. Mannitol egg yolk polymyxin (MYP) agar (Difco) was utilized to estimate the probiotic concentrations in the feed (cfu/g) [25]. Weekly feeds were meticulously prepared to maintain a consistent concentration of probiotics in the diets.

### 2.5. Experimental Setup and Fish Management

Following a period of probation, 270 fish of uniform size (weighing 27.2 ± 1.7 g and 9.0 ± 1.2 cm in length), which were healthy and free from diseases, were divided into three groups: a control group (GC) with no probiotic inclusion and two groups with probiotic inclusion diets at the concentrations of 10^6^ cfu/g (G1) and 10^8^ cfu/g (G2), respectively. Nine plastic tanks were used, with each tank containing 30 fish, each group was kept with triplicates. The fish were fed three different diets: commercial feed GC (without probiotics), G1 (with probiotics at a concentration of 10^6^ cfu/g), and G2 (with probiotics at a concentration of 10^8^ cfu/g). The trial period lasted 30 days, the fishes were given a commercial diet or commercial diet-supplemented probiotics twice daily at 08:30 and 16:30, amounting to 5% of their body weight [22]. Measurements of fish weight on a weekly basis were conducted to assess their growth and to make any necessary adjustments to the daily feeding ratio. To ensure more precise feed conversion ratio (FCR) values, we measured the weight of the feed before each feeding session to ensure consistent feed intake. The diets were carefully prepared weekly to ensure optimal results. Throughout the trial duration, water was consistently replaced, and undigested feeds were frequently sucked out using the siphoning method.

### 2.6. Sample Collection

#### 2.6.1. Growth Performance

The fishes were not fed for 24 hr before the end of the feeding trial. After completing the feeding experiment, each treatment group, consisting of 15 similarly sized fishes, was administered with the anesthetic MS-222 (100 mg/L) to make them unconscious before sample collection. The fish's growth performance, including final weight (Wt), weight gain rate (WGR), survival rate (SR), specific growth rate (SGR), and FCR, was calculated as follows: Weight gain rate (WGR, %) = (final fish weight (g)−initial fish weight (g))/initial fish weight (g) × 100, survival rate (SR, %) = 100 × final fish number/initial fish number, specific growth rate (SGR%) = 100 × ln(final fish weight (g))−ln(initial fish weight (g))/ time of the experiment covered, and feed conversion ratio (FCR) = total feed intake (g)/(final fish weight (g)-initial fish weight (g)), similar to the method described by Xue et al. [20].

#### 2.6.2. Mucus and Serum Sampling

Skin mucus was collected using the methodology described by Gobi et al. [18]. Briefly, mucus was collected from the front to the back and mixed with tris-buffered saline (TBS), a solution containing 50 mM tris-HCl and 150 mM NaCl, at pH 8.0. The homogenate was centrifuged at 1,500 *g* for 10 min at 4°C. The resulting liquid above (supernatant) was preserved at −80°C. Following a 30-day feeding experiment, six fishes were randomly selected from each tank and subjected to 24 hr food deprivation. The fishes were immobilized using MS222 (Sigma-Aldrich, Louis, MO, USA) at 100 mg/L. Blood from each fish was extracted from the caudal vein using a 1 mL syringe and transferred into plastic Eppendorf tubes. The tubes were refrigerated at 4°C for 12 hr, after which the blood samples were centrifuged at 3,000 *g* for 10 min. This process resulted in serum extraction, and the collected serum was preserved at −80°C [18]. According to a study by Gobi et al. [18], immunological markers and antioxidant enzyme activities can be assessed using serum and mucus. Tissue samples were obtained from the distal intestines of six randomly selected fishes from each tank. The distal tissue was cleaned using saline solution (0.65%) on a sterile operating table. It was then transferred to microcentrifuge tubes and immediately submerged in liquid nitrogen (LN_2_).Finally, the tissue was stored at −80°C for further metabolomics investigation on the gastrointestinal tract [26].

### 2.7. Immunological Markers and Biochemical and Antioxidant Indices

#### 2.7.1. Lysozyme (LYZ) Activity

We used the technique of Liu et al. [27] to evaluate LYZ activity in skin mucus and serum. In summary, we utilized commercially available assay kits from the Nanjing Jiancheng Institute in Nanjing, China, to quantify LYZ levels. LYZ activity was determined using a spectrophotometer (UV-6100; Shanghai Yuanxi Instrument Ltd., Shanghai, China) at a wavelength of 530 nm. Units per millilitre of serum or mucus per protein was used to determine the LYZ activity.

#### 2.7.2. Reactive Oxygen Species (ROS) Activity

Intracellular respiratory burst activity, which involves the release of superoxide anions, was assessed by assessing the formation of ROS using the technique described by Rubio and Cerón [28]. Optical density was measured at 540 nm using a plate reader.

#### 2.7.3. Reactive Nitrogen Species (RNS) Activity

The Griess reagent technique, which quantifies nitric oxide (NO) by converting it to nitrite, was used to assess NO in serum and skin mucus [29]. The concentration of nitrite in the culture medium was determined by utilizing a standard curve.

#### 2.7.4. Superoxide Dismutase (SOD) Activity

The SOD activity was quantified using the technique described by Gobi et al. [18]. The variation in absorbance was quantified at 560 nm (*∆A*560). The SOD activity was quantified as U mg/protein.

#### 2.7.5. Glutathione Peroxidase (GPx) Production

GPx activity was meticulously measured using the technique described by Dworzański et al. [30], which involves precisely measuring the oxidation of NADPH in the presence of H_2_O_2_ at a wavelength of 340 nm. GPx activity was quantified with the highest accuracy as nmol NADPH/min mg per protein.

#### 2.7.6. Protein Analysis

The dye-binding method, a reliable and widely accepted technique, was employed to determine the protein content of serum and skin mucus, with bovine serum albumin (BSA, Sigma) as a reference, following the procedure described by Ghafoori et al. [31].

#### 2.7.7. Alkaline Phosphatase (ALP) Production

ALP activity was measured using the technique described by Tang et al. [32]. A unit of activity was expressed as the quantity of enzyme needed to produce 1 mmol of the p-nitrophenol product in 1 min.

#### 2.7.8. Myeloperoxidase (MPO) Analysis

The method described by Pulli et al. [33] was used to measure the activity of MPO in skin mucus and serum. A unit was defined as the quantity that resulted in a change in absorbance of one, and the production was represented as units per milligrams mucus or serum per protein.

### 2.8. Metabolomics

#### 2.8.1. Intestine Preparation for Metabolite Examination

After the dissection of the samples into tiny fragments and the removal of fat-rich tissues, a solution of phosphate-buffered saline (PBS) was used to clean the gastrointestinal tract and remove any traces of blood. The intestinal fragments were first stored in LN_2_ and then moved to a freezer set at −80°C for future examination. After thawing, the tissues were mixed with pure water until they were completely homogenized (100 mg of tissue dissolved in 500 *μ*L of ultrapurified H_2_O). The intestinal fragments were removed from the electropolish (EP) tube using 300 mL of CH₃OH. After a 10-min ultrasound, 20 L of normal internal material was extracted, followed by vigorous mixing for 30 s. The intestines were incubated at 20°C for 1 hr for further elimination of all proteins. After centrifugation (13,000 rpm, 15 min, 4°C), the liquid portion (supernatant) was transferred to a glass container for mass spectrometry (LC/MS) analysis. Additionally, 2 mL of fresh liquid chromatography was added to the sample [34]. For quality control, 20 *µ*L taken ffrom each sample was used. Ultra-high-performance liquid chromatography quadrupole time-of-flight mass spectrometry (UHPLC-QTOF-MS) analysis was performed using 180 *µ*L of the supernatant [35].

#### 2.8.2. Metabolites Extraction and (UHPLC)-LC-MS Analysis

LC-MS analysis was conducted using state ultra-high-performance liquid chromatography (UHPLC). We used a TripleTOF 5,600 mass spectrometer, a cutting-edge instrument, in conjunction with a UPLC BEH amide column, to ensure the highest level of accuracy. The mobile phase was then prepared. The mobile phase was composed of a solution containing 25 mM NH_4_OAC and 25 mM NH_4_OH in water (pH value of 9.75) along with acetonitrile. The gradient elution technique was used at a flow rate of 0.5 mL/min. The procedure consisted of the following steps: starting with 2% B for 1.5 min, 2%–100% B for 12 min, 100% B for 14.0 min; 100%–2% B for 14.1 min, and finally returning to 2% B for 17 min, where B is methanol (0.1% (v/v) formic acid water). The injection was administered in a volume of 3 *µ*L. LC-MS analysis was performed using triple time-of-flight (TOF) mass spectrometry and information-dependent (IDA) MS/MS spectrometry. Imaging was initiated by activating the tandem hold using the mass spectrometry (MS/MS) spectrum after all scan surveying programs were executed with different preselected configurations. Throughout the process and evaluation, the MS data were not interrupted. Each loop was designed to fragment 12 precursor ions, with a collision energy of 30 V.

#### 2.8.3. Metabolite Identification and Differential Analysis

Thévenot et al. [36] identified Q2, R2Y, and R2X as the measurement indices for orthogonal fractional least squares discriminant determination (OPLS-DA). The translation amounts of the *X* and *Y* matrices of the constructed model are denoted as R2X and R2Y, respectively, and the model's predictive ability is denoted as Q2. An accurate model was projected to have Q2 > 0.5, while an exceptional model was expected to have a Q2 value greater than 0.9. Ensuring alignment authentication is crucial for assessing dependability of the OPLS-DA model. Owing to its considerable importance, the model was maintained in its current state. When the Q2 and R2 values were equal to or lower than the variance metabolites, they were selected using the factors vital in the prediction (VIP) analysis. Furthermore, the VIP value of the OPLS-DA model, *p* value of the *t*-test, and method of combining the variance multiples were implemented to enhance the specific metabolites of biological replicates [37]. We used VIP > 1.5, *p*=0.01, and fold change (FC) > 3 as selection standards. The annotated metabolites utilized in this study were obtained from Kyoto Encyclopedia of Genes and Genomes (KEGG). This database plays a pivotal role in facilitating comprehensive analysis of metabolite content, expression data, and genes [36]. Metabolomics pathway analysis (MetPA) was employed in conjunction with the KEGG to process the metabolite pathways. Improvement in metabolic routes by differential metabolites was investigated using the KEGG database. Metabolic routes were considered enriched when *x*/*n* > *y*/*N* was reached. Significant enrichment of metabolic pathways was determined when the *p* value was <0.05. To prepare the data for heatmap clustering using differential metabolite intensity regions, a *Z*-value was used to standardize the results. Subsequently, it was visualized using a heatmap tool in R software. The analysis of chord plots was improved using the R package GO plot 1.0.2 [38].

### 2.9. Statistical Analysis

Our statistical analysis involved several tests. To examine the differences between groups, one-way analysis of variance (ANOVA) was conducted when the data variance was homogeneous. Differences between treatment groups were considered statistically significant at *p* < 0.05, using Tukey's honest significant difference (HSD) tests, and data were presented as mean ± SD. Receiver operating characteristic (ROC) curve analysis was conducted for metabolites to determine the area under curve (AUC), which compares the predictive ability of metabolites. Furthermore, we created graphical depictions of immune responses using GraphPad software LLC (version 9.0; San Diego, California).

## 3. Results

### 3.1. Growth Performance

As shown in Table 2, Wt, WGR, SR, and SGR of fish fed G2 were significantly higher than those fed G1 and the control diet (GC). There was no notable difference in the FCR for diets given to fish between G2 and G1. Fish that were fed the GC had a notably greater FCR (*p* < 0.05).

### 3.2. Biochemical Parameters

**Figure 1:**
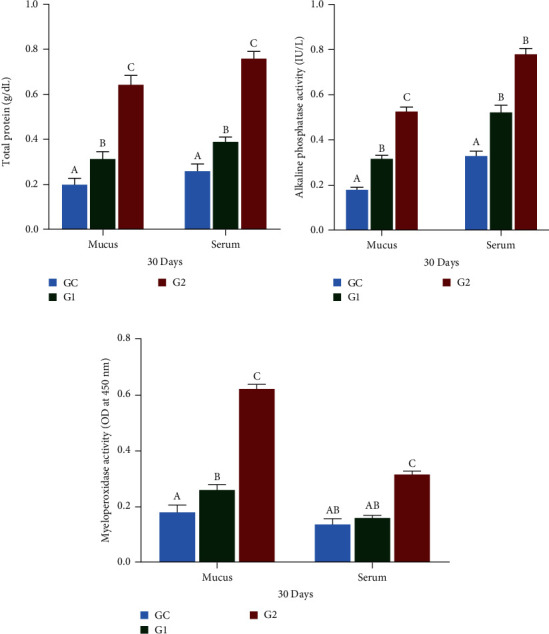
TP (a), ALP (b), and MPO (c) in mucus and serum of *O. niloticus*. Control: fish fed without probiotics (GC). G1 and G2: with commercial diet containing *B. amyloliquefaciens* AV5 at 10^6^ and 10^8^ cfu/g diet, respectively. TP denotes total protein, ALP denotes alkaline phosphatase, and MPO denotes myeloperoxidase activity. The values are shown as mean ± S.D of three replications. Based on Tukey's test, values with distinct letters on the same line show a significant difference (*p* < 0.05).

Figure 1(a) shows that after 30 days, the experimental diet groups had significantly higher protein concentrations in mucus and serum compared to the control group (*p* < 0.05). The G2 group showed the highest protein levels. Both G1 and G2 groups also had significantly higher ALP levels in the serum and mucus (*p* < 0.05) than the control group, with serum showing a more pronounced response. The G2 group had the highest ALP level, whereas the control group had the lowest level. Additionally, the G1 and G2 groups exhibited significantly higher MPO activity in skin mucus than the control group (*p* < 0.05). The G2 group showed the highest MPO activity in both the serum and mucus.

### 3.3. Immune Response Markers

**Figure 2:**
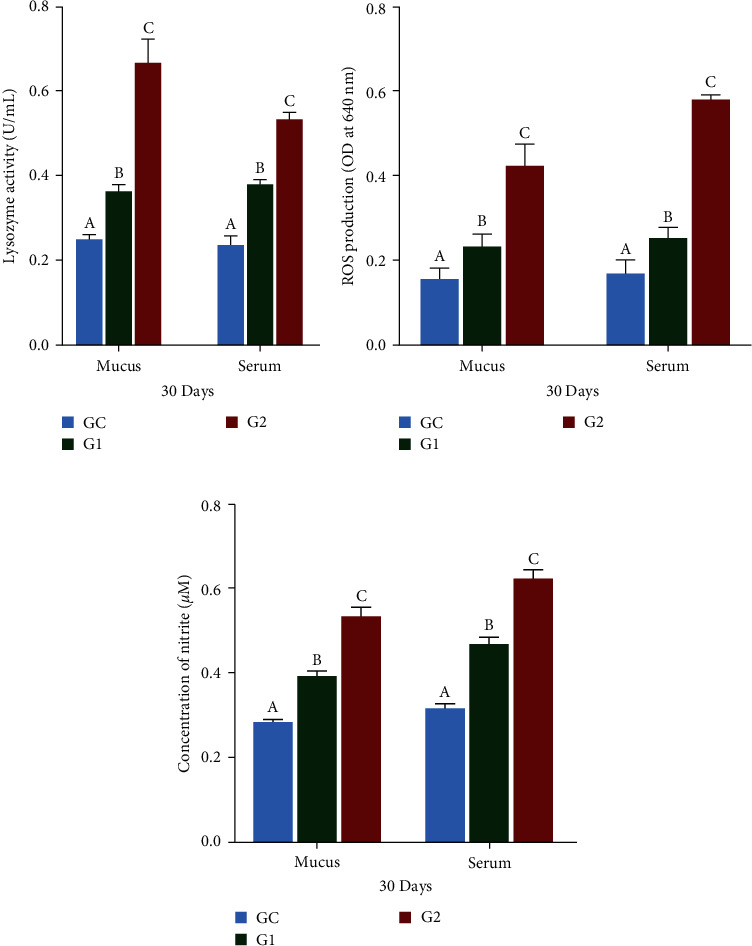
LYZ (a), ROS (b), and RNS (c) in mucus and serum of *O. niloticus*. Control: fish fed without probiotics (GC). G1 and G2: with commercial diet containing *B. amyloliquefaciens* AV5 at 10^6^ and 10^8^ cfu/g diet, respectively. LYZ denotes lysozyme activity, ROS denotes reactive oxygen, and RNS denotes reactive nitrogen activity. The values are shown as mean ± S.D of three replications. Based on Tukey's test, values with distinct letters on the same line show a significant difference (*p* < 0.05).

After 30 days of treatment, LYZ levels in the serum and skin mucus of Nile tilapia significantly increased in the G1 and G2 groups compared to the control group (*p* < 0.05), as shown in Figure 2(a). The G2 group had the highest LYZ levels in both the serum and skin mucus. Skin mucus showed greater LYZ activity than serum.

### 3.4. Antioxidant Indices

**Figure 3:**
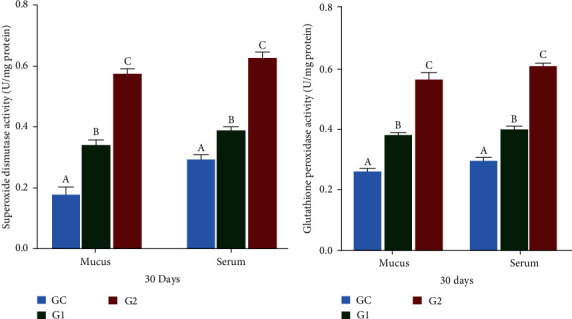
SOD (a) and GPx (b) in mucus and serum of *O. niloticus*. Control: fish fed without probiotics (GC). G1 and G2: with commercial diet containing *B. amyloliquefaciens* AV5 at 10^6^ and 10^8^ cfu/g diet, respectively. SOD denotes superoxide dismutase activity and GPx denotes glutathione peroxidase. The values are shown as mean ± S.D of three replications. Based on Tukey's test, values with distinct letters on the same line show a significant difference (*p* < 0.05).

After 30 days of feeding, the generation of ROS in the serum and mucus substantially increased (*p* < 0.05) in the G1 and G2 groups relative to the control group (GC), as shown in Figure 2(b). ROS levels were higher in the serum than in the skin mucus, with the G2 group showing the highest levels and the GC the lowest levels. Similarly, the synthesis of RNS was significantly increased (*p* < 0.05) in the G1 and G2 groups relative to the GC group (Figure 2(c)). RNS production was also higher in serum than in skin mucus, with the G2 group exhibiting the highest levels and the GC group the lowest. SOD and GPx in the serum and mucus of Nile tilapia were significantly increased (*p* < 0.05) in the G1and G2 groups compared to those in the GC group, as shown in Figures 3(a) and 3(b). The G2 group exhibited the highest levels of SOD and GPx activities, followed by G1 and GC.

### 3.5. Metabolomics

#### 3.5.1. Principal Component Analysis (PCA) and OPLS-DA Response

**Figure 4:**
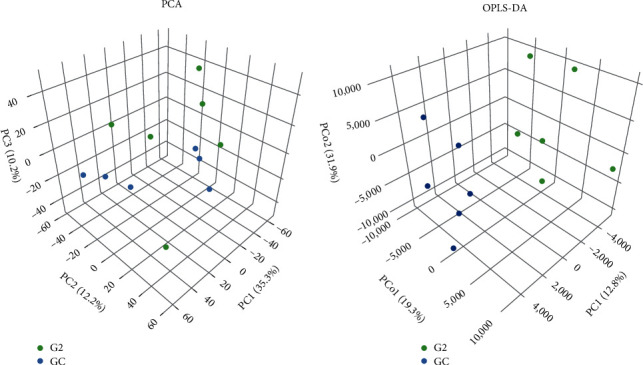
(a) Principal component analysis of LC-MS metabolite profiles (R2 = 0.942 and Q2 = 0.1094) and (b) a pairwise evaluation utilizing an OPLS-DA score graph (Q2 = 0.78, R2Y = 0.995, and R2X = 0.722) in the Nile tilapia GC and G2 groups. GC, control diet group with no probiotics and G2, with 10^8^ cfu/g of *B. amyloliquefaciens* AV5.

PCA of the intestinal metabolites in the G2 and GC control groups, as shown in Figure 4(a), effectively differentiated the two groups with no overlap. The permutation test showed high predictability and repeatability, with R2 and Q2 values of 0.942 and 0.1094, respectively. The OPLS-DA score diagram also indicated a distinct separation between the GC and G2 groups, with a legitimate permutation test yielding R2Y and Q2 values of 0.995 and 0.78, respectively, in positive mode (Figure 4(b)). All the sample groups fell within the 95% Hotelling's T2 ellipse, with the OPLS-DA model showing Q2 = 0.78, R2*Y* = 0.995, and R2*X* = 0.722.

#### 3.5.2. Glycolysis/Gluconeogenesis and Lipid Metabolism

**Figure 5:**
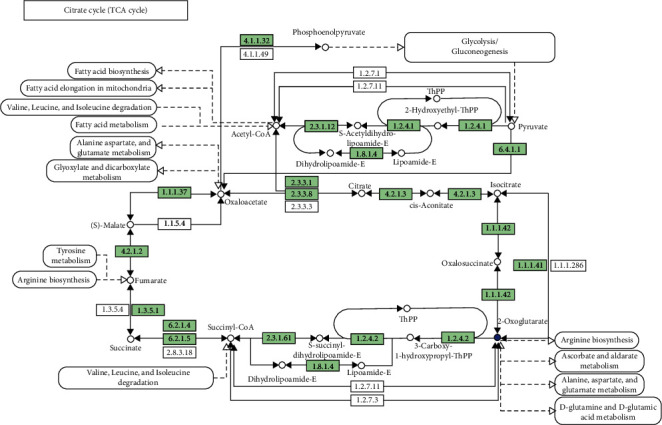
The differentially expressed genes in the gluconeogenesis/glycolysis routes are annotated in the KEGG database in Nile tilapia. GC, control diet group with no probiotics and G2, with 10^8^ cfu/g of *B. amyloliquefaciens* AV5.

**Figure 6:**
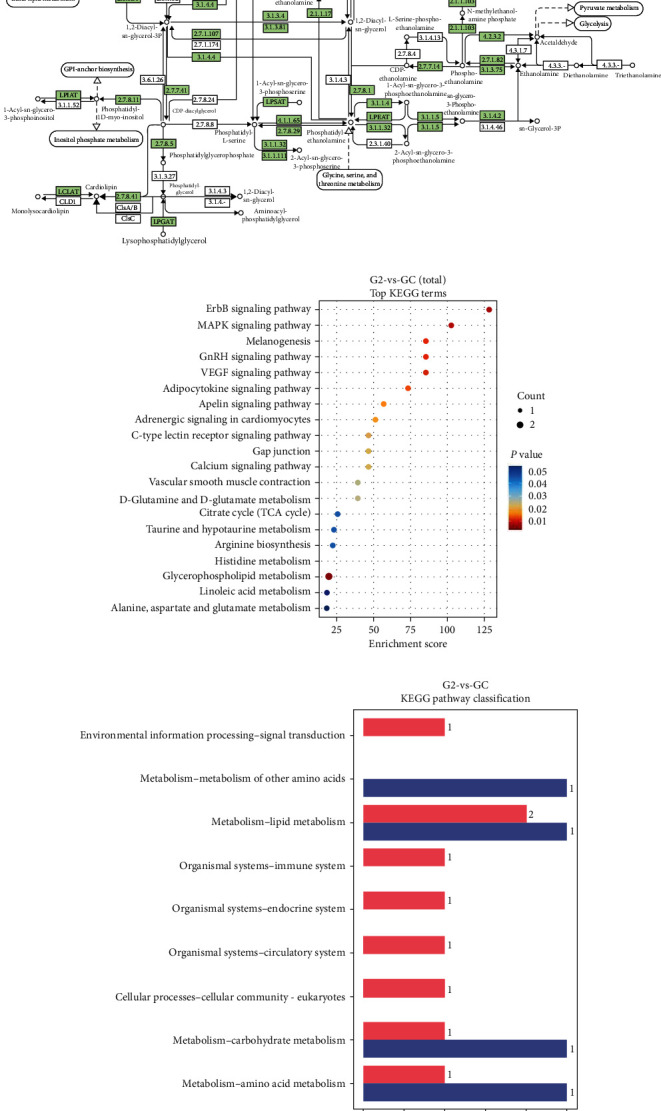
The Kyoto Encyclopedia of Genes and Genomes (KEGG) showing (a) lipid metabolism/glycerophospholipids route that differentially expressed genes, (b) enrichment of differential metabolites routes, and (c) classification of all the assembled unigenes in Nile tilapia commercial fed diet included with *B. amyloliquefaciens* AV5. GC, control group with no probiotics and G2, with 10^8^ cfu/g of *B. amyloliquefaciens* AV5.

Figure 5 illustrates the relationship between glycolysis/gluconeogenesis and the related pathways. The metabolic response was influenced by the addition of *B. amyloliquefaciens* AV5 to the diet of *O. niloticus* according to the KEGG. The inclusion of *B. amyloliquefaciens* AV5 activated many differentially expressed genes (DEGs) related to glycolysis. In addition, lipid digestion and absorption were significantly modulated in *O. niloticus*.  Figure 6(a) illustrates the complex interrelationships between lipid metabolism and the corresponding physiological mechanisms. After supplementing *O. niloticus* meal with *B. amyloliquefaciens* AV5, an increase in the activities of pyruvate kinase, 2-hydroxyethyl-ThPP, and dihydrolipoamide dehydrogenase was observed. Our findings indicate a strong correlation between lipid metabolic routes, as demonstrated by the involvement of multiple enzymes (glycerol-3-phosphate acyltransferase, 1-acylglycerol-3-phosphate acyltransferase, and diacylglycerol acyltransferase) in both the processes.

#### 3.5.3. Differential Metabolites Response

**Figure 7:**
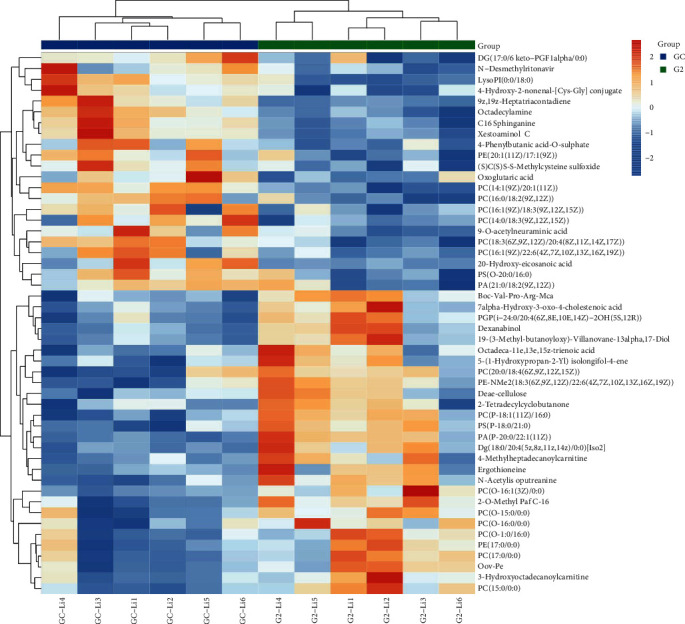
The heatmap illustrates the unsupervised hierarchical clustering in Nile tilapia, displaying the changes in metabolite levels relative to the median metabolite level. GC, control diet group with no probiotics and G2, 10^8^ cfu/g of *B. amyloliquefaciens* AV5. *Note*: The reader is directed to the Web version of this study for a description of the color references in this figure's legend.

The evaluation of various pathways for enrichment, as depicted in Figure 6(b), identified differential metabolites in 20 pathways. Histidine and glycerophospholipid metabolic pathways showed significant abundance, indicating modulation of crucial biological and cellular processes by the experimental diets (*p* < 0.05). In Figure 6(c), *O. niloticus* fed with G1 and G2 diets exhibited elevated levels of differential metabolites compared to those fed with GC diets, with the G2 group showing higher upregulation than the GC control.  Figure 7 presents a heatmap illustrating the metabolite reactions of *O. niloticus*. A notable metabolic shift was observed in the experimental groups, with higher levels of 3-hydroxyoctadecanoylcarnitine and 6-methylheptadecanoylcarnitine in the G2 group compared to the GC group.

## 4. Discussion

A probiotic-supplemented diet is an environmentally friendly preventive measure for fish health and growth in aquaculture [4]. Probiotics enhance lipid metabolism and immunity [39], and *Bacillus* strains are increasingly being used because of their significant probiotic benefits [2]. Dietary components affect the microbiota and its metabolites, and probiotics alter the composition of the gut microbiome to positively influence host health [2]. Metabolomics is a powerful method for demonstrating metabolic changes induced by probiotic ingestion in host tissues, organs, and biofluids. In contrast, lipidomics examines lipids in various cells and biofluids [40]. Limited research has explored the effects of *Bacillus* probiotics added to the diets of *O. niloticus* on lipid metabolism and metabolites [41]. The present investigation found that supplementing *O. niloticus* diets with *B. amyloliquefaciens* AV5 significantly increased Wt, WGR, SR, and SGR. The probiotic group (G2) showed the most favorable outcomes (Table 2). The greater Wt and WGR in the *B. amyloliquefaciens* treatments may be due to nutrient consumption, as indicated by the significantly reduced FCR in these groups. Comparable research has shown that *Bacillus cereus* greatly enhances the growth performance of crustaceans [41]. Numerous *Bacillus* species can enhance host growth, feed efficiency, and weight gain [18]. Supplementing Nile tilapia diets with *Bacillus licheniformis* improves growth and feed utilization [24]. *B. amyloliquefaciens* also boosts growth in *O. niloticus* and yellow catfish [20, 42]. The probiotic *B. licheniformis* enhances growth in Mozambique tilapia [18]. Probiotics promote fish weight gain by improving intestinal morphology, appetite, vitamin synthesis, and digestion [20]. Probiotics can also enhance the immune system by adhering to and colonizing fish intestines [43, 44]. Further studies are required to determine the effect of probiotics, including *B. amyloliquefaciens*, on the digestive enzyme activity of fish. Probiotic bacteria interact with the immune system through microbe-associated molecular patterns (MAMPs) such as polysaccharides (CPs), peptidoglycan (PGN), lipoteichoic acids, and lipoprotein [43]. Pattern recognition receptors (PRRs), such as nucleotide oligomerization domain (NOD)-like receptors (NLRs), toll-like receptors (TLRs), and C-type lectin receptors (CLRs), enable immune cells to bind MAMPs [43, 45]. This finding lends credence to the idea that supplementing fish feed with a live culture of *B. amyloliquefaciens* maintains high probiotic levels and boosts immune response. Probiotics improve immunological responses by interacting with the immune cells. *Labeo rohita*, *Cyprinus carpio*, and *O. mossambicus* showed higher mucus and serum protein levels when fed *B. subtilis* KADR1, *Lactococcus lactis*, and *B. licheniformis* Dahb1, respectively [18, 46, 47]. Similarly, *O. niloticus* fed with *B. amyloliquefaciens* AV5 exhibited elevated mucin and serum protein levels. Key defense proteins, such as agglutinins, lectins, LYZ, and immunoglobulins, are linked to serum and mucus proteins [18]. These findings support the development of probiotic-based fish feed to enhance health and immune responses in aquaculture and indicate that *B. amyloliquefaciens* AV5 can increase serum and mucus protein levels in fishes. In Persian sturgeon, a diet with *B. licheniformis* increased serum and mucus ALP activity [48]. *O. niloticus* showed elevated ALP activity in both serum and mucus when fed *Lactobacillus plantarum* [49]. *Enterococcus faecium* in the diet of *Rutilus rutilus caspicus* also increased ALP activity in its mucus [50]. Similarly, our study found that *O. niloticus* had higher ALP levels in mucus and serum when supplemented with *B. amyloliquefaciens* AV5. ALP, a lysosomal enzyme, activates macrophages and serves as an antibacterial agent [18]. Neutrophils release MPO from azurophilic granules during respiratory bursts, producing hypochlorous acid to eliminate pathogenic bacteria [51]. Degranulation activates the halide synthesis pathway and releases MPO and various antimicrobial enzymes. MPO activity increased in the serum of *O. mossambicus* fed *B. licheniformis*, *L. rohita* fed *B. amyloliquefaciens*, and *Catla catla* fed *B. amyloliquefaciens* [10, 18, 21]. The mucus of *O. mossambicus* also showed increased MPO levels with *B. licheniformis* supplementation [18]. Our study found elevated MPO activity in both the serum and mucus of *O. niloticus* when fed *B. amyloliquefaciens* AV5 for 30 days. LYZ is a bactericidal peptide crucial for fish innate immunity. It stimulates phagocytes and the complement system, breaks down bacterial cell walls, and prevents biofilm formation [18, 43, 52]. LYZ levels increased in *O. niloticus* fed *B. safensis* NPUST1 at 10^6^ and 10^7^ cfu/g and *B. subtilis* WB60 at 10^7^ cfu/g [53, 54]. Our research found that Nile tilapia fed *B. amyloliquefaciens* AV5 had higher LYZ activity in the serum and mucus. Red sea bream showed increased serum LYZ activity with *B. subtilis*, and *O. mossambicus* had elevated mucus and serum LYZ levels with *B. licheniformis* Dahb1 [18, 55]. *C. catla* showed increased mucus LYZ with *B. amyloliquefaciens* FPTB16 at 10^8^ and 10^9^ cfu/g [10, 18], and *O. niloticus* had higher mucus LYZ with *B. licheniformis* [24]. Phagocytes eliminate bacterial invaders through respiratory bursts that generate ROS and inhibit pathogenic infections in fish [18]. Elevated ROS levels were observed in yellow perch fed a mixture of *Bacillus* species for 6 weeks and in Pengze crucian carp supplemented with *B. cereus* [56, 57]. Increased ROS levels were also seen in the serum and mucus of Mozambique tilapia fed *B. licheniformis* Dahb1at 10^7^ cfu/mL and *C. catla* fed *B. amyloliquefaciens* FPTB16 at 10^8^ and 10^9^ cfu/g [10, 18]. Our research showed elevated ROS levels in the serum and mucus of Nile tilapia fed *B. amyloliquefaciens* AV5. In addition to ROS activity, NO is an effector molecule involved in the immune response and is connected to activated granulocytes in fish [10]. Infections can be restricted by activating macrophages, which generate RNS [10]. NO is a key regulator of immune processes with direct antimicrobial effects [43]. Elevated serum NO levels have been observed in grass carp fed *B. subtilis* [58]. Higher NO levels were also noted in *C. catla* fed *B. amyloliquefaciens* FPTB16 at 10^8^ and 10^9^ cfu/g [10] and in *O. mossambicus*-fed *B. licheniformis* at 10^7^ cfu/mL [18]. Our research found that after 30 days of feeding *O. niloticus* diets containing *B. amyloliquefaciens* AV5, NO generation increased in both serum and skin mucus, following a pattern similar to that of oxygen radical production. During phagocytosis, vertebrates produce ROS, such as hydroxyl radicals (-OH), hydrogen peroxide (H_2_O_2_), and superoxide anions (O_2_^−^), which are highly effective germicides. Antioxidant enzymes protect cells from damage caused by ROS [59]. The most prevalent antioxidant enzymes in fishes are GPx, SOD, and catalase (CAT) [60]. *O. mossambicus* supplemented with *B. licheniformis* for 4 weeks showed increased GPx and SOD levels in serum and mucus [18]. After 8 weeks, *C. catla* fed *B. amyloliquefaciens* (10^9^ and 10^8^ cfu/g) had elevated GPx and SOD levels in serum and skin mucus [10]. *Ctenopharyngodon idella* supplemented with *B. licheniformis* also showed increased GPx and SOD expression in mucus [61]. Grass carp treated with *B. subtilis* for 42 days had higher serum SOD and GPx levels [62]. Similarly, *O. niloticus* fed *B. amyloliquefaciens* AV5 (10^6^ and 10^8^ cfu/g) for 30 days exhibited increased GPx and SOD activities in serum and mucus. Metabolomics is now used in aquaculture research to tackle the long-standing challenges related to health, nutrition, enhancement, and genetics that have plagued the aquaculture industry [40]. Metabolomic methods have been proposed for various purposes, including gene expression product analysis, biomarker identification, metabolite characterization, and variation tracking [63]. Metabolomics is one of these new omics-based methods that can quantify and analyze biomolecules and metabolites that represent an animal's reaction to both internal and external influences [40]. Metabolomics is a highly effective method for investigating metabolic changes that occur in host biofluids, tissues, and organs after the intake of probiotics [64]. In our study, we employed PCA metabolite analysis to assess the effects of supplementing Nile tilapia diets with *B. amyloliquefaciens* AV5. The results revealed distinct nonoverlapping between the control group (GC) and the G2 group (Figure 4(a)), consistent with previous findings by Yin et al. [40], indicating distinct clustering of metabolic products. OPLS-DA confirmed that intestines from both the treatment and control groups fell within the 95% ellipse of Hotelling's T2 test, suggesting suitability for evaluating distal gut metabolites postsupplementation with *B. amyloliquefaciens* AV5. In addition, OPLS-DA analysis revealed notable differences in metabolites between the treatment and control groups (Figure 4(b)). Glycolysis and gluconeogenesis are vital metabolic routes for ATP generation in cells [65].They involve multiple enzymes and substrates to combine molecules and form complex compounds [65] that are essential for energy acquisition, protein synthesis, and reproduction [40]. Glycolysis is particularly important for glucose balance, converting glucose into pyruvate to produce ATP for cellular energy [66].

Conversely, gluconeogenesis converts noncarbohydrate compounds, such as fatty acids and amino acids, into glucose [65]. Pyruvate kinase converts pyruvate into phosphoenolpyruvate (PEP), which is crucial for ATP synthesis [67]. PEP is vital for glycolysis, which is the process of converting glucose into ATP [67]. The coenzyme 2-hydroxyethyl-ThPP aids in converting pyruvate to acetyl-CoA, which is essential for the citric acid cycle and ATP production [68]. It also facilitates electron transport, thereby promoting ATP generation [69]. The dihydrolipoamide dehydrogenase enzyme is essential for both the pyruvate dehydrogenase complex and S-succinyl dihydrolipoamide and acetyl dihydrolipoamide-E, facilitating electron transport in pyruvate to acetyl-CoA conversion for increased ATP production [69]. S-acetyl dihydrolipoamide-E transfers electrons from pyruvate to NAD^+^, whereas S-succinyl dihydrolipoamide transfers electrons from NADH to CoQ, which is crucial for ATP production in the citric acid cycle [70, 71]. Nile tilapia fed diets containing G1 and G2 showed elevated levels of pyruvate kinase, 2-hydroxyethyl-ThPP, and dihydrolipoamide dehydrogenase, which enhanced the glycolytic pathway activity (Figure 5). This suggests that highly unsaturated phospholipids may protect liver cell membranes, improve mitochondrial function, promote lipid metabolism, and reduce extra fat levels in the fish [70]. To the best of our knowledge, research on metabolomics in fish using UPLC-MS is limited, with few results and no comprehensive literature review to elucidate our findings. Therefore, further investigation in this area is recommended. Glycerophospholipids are essential lipids involved in various biological processes [72]. Enzymes such as diacylglycerol acyltransferase, glycerol-3-phosphate acyltransferase, and acyl-CoA-1-acylglycerol-3-phosphate acyltransferase are key in their biosynthesis, regulating their synthesis, remodeling, and degradation to maintain membrane structure [72]. The study found that glycerol-3-phosphate acyltransferase, 1-acylglycerol-3-phosphate acyltransferase, and diacylglycerol acyltransferase are crucial for lipid metabolism and that experimental diets improved these metabolic pathways compared to the control group (Figure 6(a)). Further research is needed to examine glycerophospholipids in fish fed diets supplemented with *B. amyloliquefaciens* or other probiotics. Histidine and glycerophospholipid metabolisms are vital for numerous biological activities [73]. Histidine, an essential amino acid, is crucial for protein synthesis, histamine production, pH control, and creation of histidine-containing dipeptides and neurotransmitters [73]. Glycerophospholipid metabolism maintains proper membrane structure and function because glycerophospholipids are critical components of cellular membranes [73]. Both histidine and glycerophospholipid metabolism are essential for cellular integrity and proper functioning of various organs and systems. Additionally, histidine and glycerophospholipid metabolism has been linked to various disorders and diseases [72]. Understanding the enzymes involved in glycerophospholipid metabolism is crucial for gaining insights into the mechanisms that regulate membrane structure and function. In this study, histidine and glycerophospholipid metabolism were enriched in the experimental diets compared to the control group (Figure 6(b)). However, few reports exist on histidine and glycerophospholipid metabolism in fish following probiotic supplementation. Researchers have aimed to discern metabolic pathways from distinct metabolites to establish their relationships. This study focused on quantifying the metabolic response of Nile tilapia to commercial diets containing *B. amyloliquefaciens* AV5. The metabolic pathway study revealed that the treatment diets (G1 and G2) had a greater impact on lipid, carbohydrate, and amino acid metabolism than the control diet (GC) in Nile tilapia (Figure 6(c)). However, its effect on glucose metabolism remains unclear. Protein metabolism has been found to enhance amino acid degradation. The functions of 3-hydroxyoctadecanoylcarnitine and 6-methylheptadecanoylcarnitine are still unknown because of the limited research on these compounds in fish. Given the general role of carnitines in cellular metabolism, these compounds may be involved in lipid metabolism or energy production [74]. Optimal carnitine activity is necessary for substrate utilization in oxidative processes [74]. In this study, 3-hydroxyoctadecanoylcarnitine and 6-methylheptadecanoylcarnitine levels were higher in fish fed commercial diets supplemented with *B. amyloliquefaciens* AV5 than in those fed commercial diets alone (GC; Figure 7). The addition of *B. amyloliquefaciens* AV5 to fish diets may lead to increased levels of carnitine-associated metabolites. The results of this investigation suggest that supplementing commercial fish diets with *B. amyloliquefaciens* AV5 could potentially improve their health and lipid metabolism by modulating carnitine-related metabolites. However, further research is needed to determine the precise role of carnitines in fish physiology and metabolism.

## 5. Conclusions

In this study, the effects of adding *B. amyloliquefaciens* AV5 to commercial fish diets on immunological response (serum and mucus), metabolomics, and lipid metabolism after 30 days of feeding were assessed. The growth, immune parameters, and activities of antioxidant enzymes in the serum and mucus of *O. niloticus* were enhanced by adding 10^6^ and 10^8^ cfu/g of *B. amyloliquefaciens* AV5. Moreover, the regulatory effect on glycolysis/gluconeogenesis pathways and lipid metabolism was determined by modifying the morphology of histidine and glycerophospholipids. Probiotic administration at a dosage of 10^8^ cfu/g had the best effects in this study and should be recommended for application in aquaculture.

## Figures and Tables

**Table 1:** Composition and proximate values of the basal diets (expressed as percentage dry weight) for Nile tilapia (*O. niloticus*) fingerling.

Basal diet analysis	Raw materials and nutrient composition
Ingredients	Fish meal
	Soybean meal
	Peanut meal
	Rapeseed meal
	Flour
	Calcium dihydrogen phosphate
	Zinc sulfate
	Ferrous sulfate
	Vitamin A
	Vitamin C
	Vitamin D3
	Vitamin E
	Folic acid

Analyzed proximate composition	Crude protein ≥ 34.0%
	Crude fat ≥ 2.50%
	Crude fiber ≤ 8.00%
	Crude ash ≤ 15.0%
	Water ≤ 11.0%
	Lysine ≥ 1.80%
	Calcium 0.5%–2.50%
	Total phosphorus 0.6%–2.0%
	NaCl ≤ 2.00%

**Table 2:** Growth indexes of Nile tilapia after 30 days of feeding with a commercial feed as a control diet (GC) and a diet supplemented with *B. amyloliquefaciens* AV5 at 10^6^ cfu/g and 10^8^ cfu/g as G1 and G2, respectively.

Growth indexes	GC	G1 (10^6^ cfu/g)	G2 (10^8^ cfu/g)
Wi (g)	23 ± 0.3	23 ± 0.4	23 ± 0.2
Wt (g)	42 ± 2.4^a^	46 ± 1.5^a^	55 ± 1.25^b^
WGR (%)	82.2 ± 1.3^a^	104.3 ± 1.4^a^	139.1 ± 1.2^b^
SGR (%)	1.42 ± 0.8^a^	1.68 ± 0.4^a^	2.07 ± 0.2^b^
FCR	1.18 ± 0.1^a^	1.02 ± 0.3^a^	0.67 ± 0.2^b^
SR (%)	98.19 ± 2.94	98.67 ± 1.92	99.98 ± 1.04

*Note*. Results are presented as the mean ± S.D (*n* = 3). Based on Tukey's test, values with distinct superscripts on the same line show a significant difference (*p* > 0.05). Thus, the following variables are defined: initial weight (Wi), final weight (Wt), weight gain rate (WGR), specific growth rate (SGR), feed conversion ratio (FCR), and survival rate (SR).

## Data Availability

Data will be provided upon request.
